# Simply adding oral nutritional supplementation to haemodialysis patients may not be enough: a real-life prospective interventional study

**DOI:** 10.3389/fnut.2023.1253164

**Published:** 2023-10-19

**Authors:** Andreja Ocepek, Robert Ekart, Petra Povalej Bržan, Sebastjan Bevc

**Affiliations:** ^1^Department of Gastroenterology, Clinic for Internal Medicine, University Medical Centre Maribor, Maribor, Slovenia; ^2^Department of Dialysis, Clinic for Internal Medicine, University Medical Centre Maribor, Maribor, Slovenia; ^3^Faculty of Medicine, University of Maribor, Maribor, Slovenia; ^4^Faculty of Electrical Engineering and Computer Science, University of Maribor, Maribor, Slovenia; ^5^Department of Nephrology, Clinic for Internal Medicine, University Medical Centre Maribor, Maribor, Slovenia

**Keywords:** haemodialysis, protein-energy wasting, oral nutritional supplementation, serum albumin, phase angle, hand-grip strength test

## Abstract

**Introduction:**

Protein-energy wasting (PEW) is a common and serious co-morbidity in haemodialysis (HD) patients. Its importance as a prognostic factor has been increasingly recognised during the past decades. Much effort has been invested in the improvement of nutritional status and amelioration of consequences through different therapeutic approaches, either intradialytic parenteral nutrition or more commonly oral nutritional supplementation. In the article, we present the results of a prospective study in HD patients after 12 months of therapeutic intervention with oral nutritional supplements (ONS).

**Methods:**

A total of 92 HD adult patients were enrolled in the study after 3 months of wash-out period. Baseline nutritional status was assessed using composite scores, laboratory markers, bioelectrical impedance analysis, and hand-grip strength test. Patients recognised as undernourished or at high risk for undernutrition received renal-specific commercially available ONS on HD day in addition to their regular diet. After 12 months, the effect of ONS on surrogate markers of undernutrition, serum albumin level, phase angle, and hand-grip strength was analysed in 71 surviving patients.

**Results:**

After 12 months, data for 71 patients, 39 (54.9%) men, 62.4 ± 12.9 years, and median haemodialysis vintage 53.3 (IQR 27.5–92.8) months, were available. Patients were divided into three groups: group A patients were with normal nutritional status at baseline not necessitating ONS; group B patients received ONS; and group C patients were entitled to receive but refused to take ONS. The baseline results showed statistically significant differences between the groups in serum albumin levels and phase angle but not hand-grip strength. Differences between the groups remained statistically significant at month 12; we did not find any statistically significant positive changes within the groups, indicating no positive effect of intervention with ONS.

**Conclusion:**

In a prospectively designed interventional single-centre study, we did not find a statistically significant change in surrogate markers of PEW in our cohort of HD patients, receiving ONS for 12 months. Since PEW is an independent risk factor influencing the survival of HD patients, efforts should be directed towards a timely and comprehensive nutritional approach, including intensive, personalised dietary counselling, increase in protein and energy intake and advocating tight control of nutritional status during HD treatment, possibly providing psychological support and motivation.

## Background

Protein-energy wasting (PEW) is highly prevalent in patients with chronic kidney failure, especially in patients undergoing maintenance haemodialysis (HD) (1). PEW is a complex syndrome influenced by multiple factors. Insufficient dietary energy and protein intake and chronic inflammation and catabolic effects of HD procedure are just some of many underlying issues that impact the nutritional status (NUS) of HD patients. Standard PEW criteria developed in 2008 by the International Society of Renal Nutrition and Metabolism (ISRNM) are time-consuming and difficult to implement in daily clinical practise; therefore, simplified classifications or surrogate markers of malnutrition may be more applicable in day-to-day work (2, 3). Although non-specific and influenced by many different factors, low serum albumin remains an important indicator of malnutrition and a simple yet strong predictor of mortality risk in HD patients (4, 5). Other biochemical parameters, e.g., serum lipids, total iron binding capacity (TIBC), and C-reactive protein (CRP), are readily used as malnutrition-inflammation markers, while serum potassium and phosphorus levels are monitored safety parameters in HD patients (4, 5). Among anthropometric measurements, body mass index (BMI) and mid-upper arm muscle circumference (MUAMC) calculated from mid-arm circumference (MAC) and triceps skinfold thickness (TSFT) are regularly utilised, as hand-grip strength (HGS) test for assessment of muscle function and bioelectrical impedance analysis (BIA) derived phase angle (PhA) as an indicator of body cell mass and integrity (5, 6). Nutritional intervention studies in HD patients have indicated that the addition of protein and energy-rich supplements to a regular diet may exert a positive effect on several nutritional parameters, subjective global assessment (SGA) of NUS, performance status, quality of life, and prognosis, including hospital re-admission rates and all-cause mortality (7–9). On the other hand, a meta-analysis elucidated only low-quality evidence in support of short-term (up to 6 months) oral energy or protein/amino acid supplements in improving NUS (4). Consequently, the authors concluded that further studies were necessary, particularly regarding the effects of oral nutritional supplementation (ONS) on mortality and quality of life (4).

This study aimed to assess the effect of long-term (12 months) ONS on surrogate markers of PEW in a real-life, single-centre cohort, prospective interventional study in HD patients.

## Methods

### Study design

This was a prospective, longitudinal interventional study with repeated measure design on a well-defined cohort of HD patients at the Department of Dialysis, University Medical Centre Maribor, Slovenia.

### Study population

**Figure 1:**
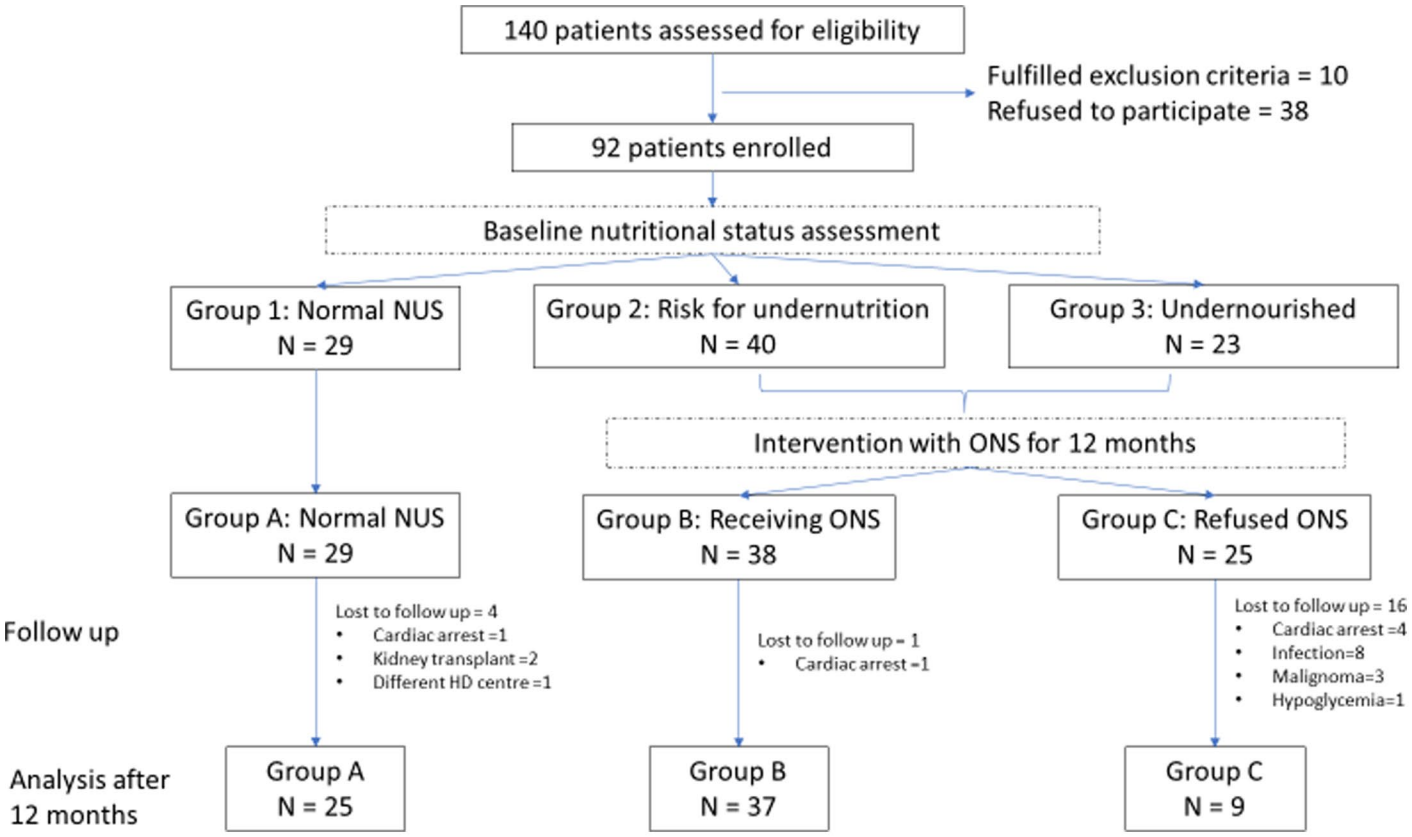
Flowchart of clinical study. NUS, Nutritional status; ONS, Oral nutritional supplements; and HD, Haemodialysis.

We assessed 140 consecutive patients for study eligibility. All patients on HD were considered. Patients younger than 18 years and undergoing dialysis for less than 6 months were excluded. Thirty-eight patients refused to participate. Finally, 92 patients were included in the study. Figure 1 shows the patient selection process.

### Measurements and variables

We collected data on the medical history along with prescribed medications, HD vintage, atherosclerosis risk factors, and co-morbidities (arterial hypertension, diabetes mellitus, dyslipidaemia, ischemic heart disease, peripheral arterial disease, cerebral arterial disease, and cancer). To perform laboratory tests, blood was drawn from a peripheral vein prior to and after HD. Patients were categorised into three groups after baseline NUS was assessed (group 1: patients with normal NUS; group 2: patients with high risk for undernutrition; and group 3: undernourished patients) using composite tools including SGA, malnutrition-inflammation score (MIS), and anthropometric and biochemical parameters. Laboratory parameters regularly used to describe malnutrition in HD patients, e.g., serum albumin, total cholesterol, triglycerides, TIBC, inflammation marker CRP, and safety markers potassium and phosphate, were recorded. Anthropometric measurements were made, e.g., body weight after the HD procedure and body height were measured with a standard medical scale; waist circumference (WC) and MAC were measured in centimetres using a tape; TSFT was measured in millimetres using Jamar® skinfold calliper (Lafayette Instrument, Lafayette, IN, United States); BMI and MUAMC were calculated using standard formulas (BMI (kg/m^2^) = body weight (kg)/body height (m)^2^ and MUAMC (cm) = MAC (cm) − 0.314 × TSFT (mm)). Additionally, body composition using BIA and HGS tests was measured at inclusion, during follow-up and after 12 months. BIA were performed after regular HD sessions with the Bodystat® Quadscan 4000 device (Bodystat Ltd., Isle of Man), with the patient resting in a supine position with pairs of electrodes placed on the dorsum of the hand and foot on the side of the body opposite to the arteriovenous (AV) fistula. The software provided by the manufacturer calculated PhA, fat-free mass index (FFMI), and dry lean mass (DLM). After the same HD session, HGS on the arm without AV fistula was measured in kilogrammes with the patient in a sitting position, their elbow at 90°, and their forearm in a neutral position using a Jamar® handgrip hydraulic dynamometer (Lafayette Instrument, Lafayette, IN, United States). The calculated average HGS of three consecutive measurements was used for analysis. Data on the dietary caloric and protein intake of patients were gathered via a 72-h recall questionnaire and assessed using PRODI® software (Nutri-Science GmbH, Hausach, Germany). In the present study, no sub-report of dietary questionnaires was analysed.

### Intervention

After a 3-month wash-out period for all subjects, undernourished patients (group 3) and patients with a high risk for undernutrition (group 2) were prescribed ONS for 12 months. In addition to their regular diet, patients received two bottles of commercially available disease-specific sip-drink (Nepro HP®, Abbott Nutrition), one to drink after every HD procedure and one to take and drink at home until the next HD day. They were asked to return empty bottles of ONS as proof of adherence. All patients received nutritional counselling from the treating physician and principal investigator, but regular clinical dietitian follow-up was not available. All patients were equally followed according to their HD schedule, and the duration of the intervention was 12 months.

### Outcome

The primary outcome was defined as the change in surrogate markers of NUS, e.g., serum albumin, PhA, and HGS, from baseline to month 12. Secondary outcomes included changes in another laboratory (TIBC, total cholesterol, triglycerides, and CRP), anthropometric (BMI, WC, MAC, and MUAMC), and BIA (FFMI and DLM) measurements and two safety parameters, such as serum potassium and phosphate concentrations.

### Statistical analysis

Statistical calculations were conducted using the R programming language (10). Numerical variables are summarised as a mean value ± standard deviation (MV ± SD) or a median and interquartile range (IQR) taken at the 25 and 75th percentiles. Categorical variables are presented as a frequency (percentage). An ANOVA or non-parametric Kruskal–Wallis test was used to compare the numerical variables among the groups. In the case of statistically significant differences among the groups, we performed Tukey’s or Dunn’s non-parametric *post-hoc* test for paired comparison. For categorical variables, Fisher’s exact test was used. Changes in observed parameters at baseline and after 12 months within groups were compared using paired samples *t*-test or non-parametric Wilcoxon signed-rank test depending on data distribution. SPSS programme v. 28.0 was used for intention-to-treat (ITT) and per protocol (PP) analyses, and analysis of covariance (ANCOVA) was run to determine the effect of the group on the post-intervention results after controlling baseline measurements. A *p* value of <0.05 was considered statistically significant.

## Results

Data for 71 patients, 39 (54.9%) men, 62.4 ± 12.9 years, and median HD vintage 53.3 (IQR 27.5–92.8) months, were available for analysis after 12 months. The most prevalent co-morbidities were arterial hypertension, diabetes mellitus, and cardiovascular diseases, contributing to the second leading cause of death in our cohort, in accordance with other authors confirming atherosclerosis as one of the most significant causes of morbidity and mortality in the developed world (11). In total, three patients were lost to follow-up (two patients received a kidney transplant, and one patient moved to another HD centre) and 18 patients died, among them eight patients died due to an infectious cause (four of pneumonia and four of sepsis), six due to cardiovascular complications, three due to malignant disease (lymphoma, kidney carcinoma, and lung carcinoma), and one due to hypoglycaemia. After 12 months, according to ONS intake, three groups were formed for statistical analysis (Figure 1): group A (*n* = 25) patients were with normal NUS at baseline, group B (*n* = 37) patients received ONS, and group C (*n* = 9) patients were prescribed ONS but refused to take them. Patients who received less than half prescribed ONS for less than 3 months were classified as group C. In group B, patients received, on average, 1531.49 kcal and 61.38 g of protein per week, in addition to their regular diet. The baseline characteristics of patients in these three groups are presented in Supplementary Table 1.1. Due to the high dropout rate in group C, we first performed an ITT statistical analysis, where groups B and C are combined into group B*, and then a PP analysis, where we excluded patients of group C from the analysis who did not take ONS (Table 1).

**Table 1:** Baseline characteristics—ITT and PP analyses.

	Group A	Group B* (ITT)	ITT analysis	Group B (PP)	PP analysis
	(*n* = 25)	(*n* = 46)	*p* value	(*n* = 37)	*p* value
*Demographics*					
Age (years)	58.0 ± 13.2	64.7 ± 12.3	**0.040**	65.8 ± 11.3	**0.015**
Sex [male; *n* (%)]	17 (43.6%)	22 (56.4%)	0.103	19 (51.4%)	0.193
Dialysis vintage (months)	45.4 (19.2–89.7)	58.3 (30.4–93.4)	0.603	61.9 (30.3–85.7)	0.649
*Co-morbidities*					
Diabetes mellitus	9 (36.0%)	15 (32.6%)	0.773	12 (32.4%)	0.771
Arterial hypertension	21 (84.0%)	40 (87.0%)	0.733	32 (86.5%)	1.000
Cardiovascular diseases	22 (88.0%)	44 (95.7%)	0.337	35 (94.6%)	0.385
Cancer	1 (4.0%)	11 (23.9%)	**0.046**	10 (27.0%)	**0.038**
*Dietary parameters*	**Group A (*n* = 21)**	Group B* (*n* = 33)		**Group B (*n* = 27)**	
Dietary energy intake (kcal/kg/day)	18.2 (14.0–20.5)	21.5 (15.9–27.6)	**0.030**	21.5 (16.2–27.9)	**0.032**
Dietary protein intake (g/kg/day)	0.7 (0.5–1.0)	0.8 (0.7–1.0)	0.293	0.8 (0.7–1.0)	0.296

ITT, Intention-to-treat; PP, Per protocol; Cardiovascular diseases, ischemic heart disease, peripheral arterial disease, and cerebral arterial disease. Data are presented as *n* (%), mean ± SD, and median (25–75th). Values of *p* < 0.05 are considered statistically significant and are marked in bold.

At the baseline, groups were similar in demographics and co-morbidities. The mean age of all patients was 62.4 ± 12.9 years and was not significantly different among the groups in ANOVA analysis, but in ITT and PP analyses, patients in group A were significantly younger (*p* = 0.040 and *p* = 0.015, respectively). Patients in group B were undergoing maintenance HD for the longest period of time; however, the difference among groups was not significant (ANOVA *p* = 0.868, ITT *p* = 0.603, and PP *p* = 0.649). Patients in group B had a significantly higher prevalence of malignant disease (ANOVA *p* = 0.043, ITT *p* = 0.046, and PP *p* = 0.038). All other characteristics, e.g., gender, other co-morbidities, and dietary intake of calories and protein, were not significantly different between the groups in ANOVA analysis, but in ITT and PP analyses, dietary intake of calories was significantly lower in group A (ITT *p* = 0.030, PP *p* = 0.032; Table 1). The results of the ANOVA analysis are presented in Supplementary Table 1.1.

**Figure 2:**
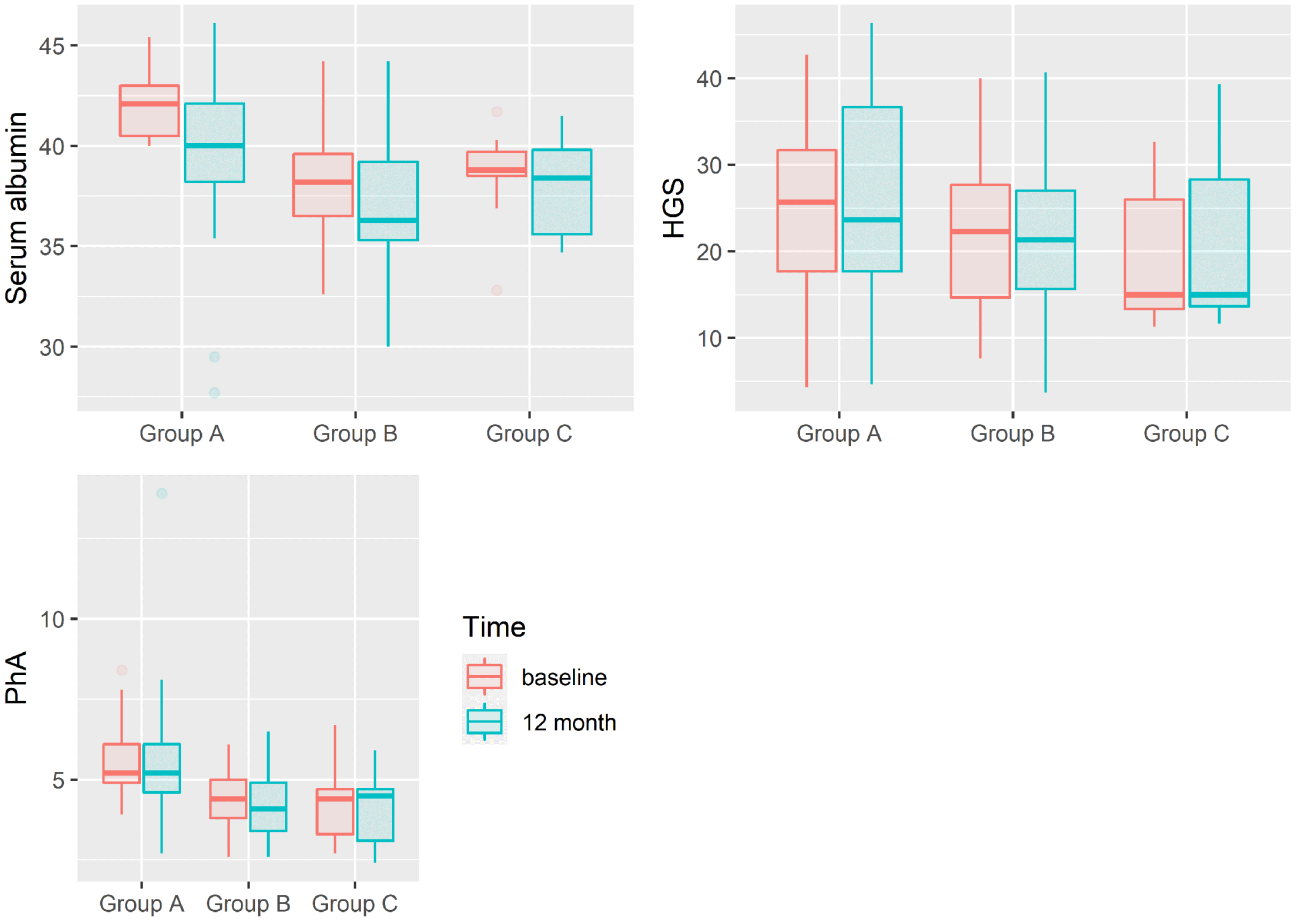
Changes in surrogate markers of PEW. PhA, Phase angle; HGS, Hand-grip strength.

According to ITT and PP analyses, changes in surrogate markers of NUS at the baseline and after 12 months within each group and between groups are presented in Tables 2, 3, respectively, and Figure 2. The baseline results showed significantly higher values in serum albumin levels (*p* < 0.001) and PhA (*p* < 0.001) when comparing group A with group B and group A with group C (serum albumin *p* < 0.001 and PhA *p* < 0.001). There were no statistically significant differences in HGS between the groups at baseline (ITT *p* = 0.166, PP *p* = 0.280). Serum albumin and PhA stayed significantly different between the groups but showed no statistically significant improvement during follow-up after 12 months within groups, including group B receiving ONS in ITT or in PP analysis, although PhA was close to reaching significance in ITT analysis. According to ANOVA (Supplementary Table 2.1), there was no statistically significant difference in HGS between and within groups during follow-up, but ITT and PP analyses together with ANCOVA adjusting for baseline values of variables within groups showed significant positive changes in HGS in group B receiving ONS (ITT *p* = 0.034, PP *p* = 0.027).

**Table 2:** Changes in surrogate markers of NUS at the baseline and after 12 months—ITT analysis.

		Group A (*n* = 25)	Group B^*^ (*n* = 46)	*p* value (between groups)	Group A (Adjusted mean)	Group B (Adjusted mean)	ANCOVA *p* value	Partial eta squared for group (measurement)
Albumin (mg/L)	Baseline	42.1 ± 1.6	38.2 ± 2.6	<0.001	39.7	39.3	0.675	0.003 (0.454)
	12 months	41.7 ± 3.3	38.2 ± 3.3	<0.001				
PhA (°)	Baseline	5.5 ± 1.1	4.4 ± 0.9	<0.001	5.2	4.4	0.054	0.054 (0.296)
	12 months	5.2 (4.6–6.1)	4.2 (3.4–4.9)	<0.001				
HGS (kg)	Baseline	24.8 ± 10.2	21.6 ± 8.5	0.166	24.8	22.2	**0.034**	0.065 (0.788)
	12 months	26.8 ± 11.8	21.1 ± 8.8	**0.043**				

PhA, Phase angle; HGS, Hand-grip strength. Data are presented as mean ± SD and median (25–75th). Values of *p* < 0.05 are considered statistically significant and are marked in bold.

**Table 3:** Changes in surrogate markers of NUS at baseline and after 12 months—PP analysis.

		Group A (*n* = 25)	Group B (*n* = 37)	*p* value (between groups)	Group A (Adjusted mean)	Group B (Adjusted mean)	ANCOVA *p* value	Partial eta squared for group (measurement)
Albumin (mg/L)	Baseline	42.1 ± 1.6	38.1 ± 2.6	<0.001	39.7	39.3	0.675	0.003 (0.454)
	12 months	41.7 ± 3.3	37.9 ± 3.5	<0.001				
PhA (°)	Baseline	5.2 (4.9–6.1)	4.4 (3.8–5.0)	<0.001	5.3	4.5	0.068	0.055 (0.255)
	12 months	5.2 (4.6–6.1)	4.1 (3.4–4.9)	<0.001				
HGS (kg)	Baseline	24.8 ± 10.2	22.1 ± 8.7	0.280	25.3	22.4	**0.027**	0.081 (0.779)
	12 months	26.8 ± 11.8	21.4 ± 8.7	**0.043**				

PhA, Phase angle; HGS, Hand-grip strength. Data are presented as mean ± SD and median (25–75th). Values of *p* < 0.05 are considered statistically significant and are marked in bold.

According to ANOVA, changes in other measured parameters at baseline and after 12 months within each group and between groups are shown in Supplementary Table 3.1. Values of BMI (*p* = 0.022 for both comparisons), MAC (*p* = 0.03 and *p* = 0.037), FFMI (*p* = 0.004 and *p* = 0.001), and DLM (*p* = 0.011 and *p* = 0.004) were significantly higher in group A when comparing it with group B and group C. MUAMC was significantly higher in group A than in group C (*p* = 0.01), but there was no statistically significant difference between groups A and B. There were no statistically significant differences in values of total cholesterol, tryglicerides, and CRP between groups at the baseline, and barely significant difference of WC and TIBC between groups A and C.

After 12 months, we observed a slight significantly positive change in FFMI (*p* = 0.039), but there are also negative changes in MAC (*p* = 0.003), MUAMC (*p* = 0.001), and DLM (*p* = 0.016) within group A and negative changes in MAC (*p* = 0.01), MUAMC (*p* < 0.001), and DLM (*p* < 0.001) within group B during follow-up. We believe that this indicates progressive muscle mass loss in HD patients, supplemented with ONS. In group C, there was no statistically significant change in any observed parameters, probably due to the small number of patients. We observed the worst outcome in group C since 16 out of 25 included patients died during 12 months of follow-up (Figure 1). In groups A and B, one patient died in each group during 12 months, both due to cardiac arrest. Although the prevalence of cancer was significantly higher in group B, no patients died due to cancer in this group. ITT and PP analyses showed that FFMI was the only measurement that was significantly different after 12 months in groups B* and B (*p* = 0.015 and *p* = 0.018, respectively). The results are presented in Supplementary Tables 3.2, 3.3.

Regarding the safety of ONS, we observed no statistically significant change in potassium and phosphate at month 12 in group B.

## Discussion

In our real-life single-centre cohort study, we prospectively evaluated the effect of ONS on surrogate markers of NUS in HD patients. According to ANOVA, we did not find any statistically significant improvement after 12 months of intervention, although our preliminary results of short-term intervention suggested a possible positive effect of ONS on the preservation of NUS in HD patients (12). Data from two systematic reviews and a meta-analysis of randomised controlled trials showed mixed results regarding the effects of ONS on certain markers of NUS in HD patients, e.g., albumin, pre-albumin, MUAMC, TSFT, BMI, and lean mass (4, 5). Although albumin is not solely a marker of NUS and is influenced by many factors in HD patients (e.g., inflammation, overhydration, and protein catabolism during HD session), it is still widely used as a traditional biochemical marker of NUS (13, 14). Albumin has also persistently been correlated with morbidity and mortality in HD patients (8, 15, 16). In our study, serum albumin at baseline was significantly lower in groups B and C than in group A, but there was no difference between groups B and C. During follow-up, we observed continuous statistically significant differences in serum albumin between groups but we observed no change in serum albumin within group B, although patients received ONS. Changes remained non-significant in ITT and PP analyses. There was a significant worsening of serum albumin in group A, where patients were evaluated as having normal NUS at baseline, indicating likely an important deterioration of overall health and NUS among these patients during a 12-month period. PhA is derived from bioelectrical impedance analysis of body composition and reflects body cell mass and integrity. PhA has been shown to be altered in patients with chronic kidney disease of different stages (17). In HD patients, PhA correlates with certain nutritional markers, e.g., albumin, pre-albumin, fat-free mass, and MUAMC, and it has been shown to be a strong independent predictor of PEW (6, 18). In our cohort of HD patients, we observed statistically significant differences in PhA between the groups at baseline, consistent with their NUS. However, PhA has worsened at month 12 in group B, despite receiving ONS. PP analysis showed a slight improvement in PhA in group B, but it was not statistically significant. The results are pointing to an important deterioration of body cell mass and integrity in this well-defined population of patients. Muscle function can, in clinical practise, be easily assessed using the HGS test. It is a simple, readily repeatable, reliable, non-invasive tool that has been shown to have a negative correlation with frequently used nutritional screening tools (e.g., SGA, MIS, and BMI) and a positive correlation with other nutritional markers (e.g., PhA, fat-free mass, and MAC) (19–21). HGS was also confirmed to be an independent predictor of all-cause mortality with a cutoff value of 22.5 kg for men and 7 kg for women, independent of dialysis modality (20). Our study did not demonstrate a significant difference in HGS between the groups at the baseline. We observed a statistically significant change in HGS during follow-up in group B receiving ONS, using ITT and PP analyses and adjusting for baseline measurements with ANCOVA. Furthermore, ITT and PP statistical analyses showed that patients who received ONS and were prescribed ONS but refused to take it were older and had a higher prevalence of cancer. Especially, the latter requires further careful evaluation and monitoring.

Our results partially align with the published literature, showing a positive effect of ONS on markers of NUS (1). On the other hand, a recent review in the Cochrane Library and a meta-analysis of randomised controlled trials debate that there is still a lack of evidence concerning the effect of ONS on NUS in HD patients (4, 5). We believe that the intake of calories and protein with ONS in our group of patients was not sufficient. Recommended intake is 7–10 kcal of energy and 0.3–0.4 g of protein per kg of body weight per day, which is a target for patients in our cohort (1, 22). Another important issue is the time of administration of ONS. In our study, patients received ONS after HD sessions, not intradialytically, which would expectedly counteract the catabolic effect of HD (22, 23). The reason for such an approach was the patient’s convenience and desire to avoid potential side effects, mostly intradialytic hypotension and gastrointestinal symptoms, thus lowering adherence to ONS. A critical assessment of our results proved this approach to be questionable since adherence was still insufficient, which is, in our belief, the leading cause for the negative result of the study. Up to 50% of HD patients consume less than 1.0 g of protein/kg/day with their regular diet and have insufficient dietary energy intake as well (3). In our cohort, the median dietary energy intake was 20.1 (IQR 14.8–25.1) kcal/kg/day and the median protein intake was 0.8 (IQR 0.6–1.0) g/kg/day, and patients in group A had lower baseline daily caloric intake than patients in the other two groups, presenting clearly that most patients in our cohort did not have a sufficient intake of nutrients with their regular diet, although patients did not differ in dietary intake of protein. HD patients are inclined to continue following dietary restrictions recommended during the pre-dialysis period of chronic kidney failure. Nutritional counselling is, therefore, recommended as a first intervention (23, 24). Such a basic intervention may be lacking in HD centres due to staff shortages as in our centre. As a further consequence, one of the pivotal limitations of our study was volatile adherence to ONS. In our opinion, HD patients’ low adherence to ONS is a result of taste fatigue, lack of regular dietary consultations, an inclination towards reluctance, and even depression in this group of patients. Systematic nutritional support with regular consultations with a clinical dietitian is expected to improve the NUS of HD patients, receiving ONS (25). Another limitation of our study was lack of data on intake of nutrients with regular diet after 12 months, We inferred the stability of the regular diet not taking into consideration potential reduction in intake due to ONS. Additionally, a systematic review of depression in HD patients reported high dietary non-adherence, ranging from 41.1 to as high as 98.3%, with a significant association between depressive symptoms and dietary non-adherence. The authors recommended early diagnosis and treatment of depression and close monitoring of adherence behaviour to enhance adherence and improve the quality of life and survival rates of HD patients (26). Finally, complementary and alternative medicine is also popular in the HD population, corresponding with dietary non-adherence (27, 28). Acknowledging and addressing depression and complementary and alternative medicine may significantly influence dietary adherence in our cohort of HD patients; therefore, in future, additional efforts should be directed towards psychological evaluation and support.

## Conclusion

In our cohort of HD patients, simply adding ONS to a regular diet slightly improved surrogate markers of PEW; in fact, only HGS and FFMI were significantly influenced by ONS. Taking into account many limitations of our study, we believe that further efforts must be directed towards a timely and comprehensive nutritional approach, including systematic, personalised dietary counselling to increase protein and energy intake, advocating tight control of NUS during HD treatment, and possibly providing psychological support and motivation.

## Data availability statement

The raw data supporting the conclusions of this article will be made available by the authors, without undue reservation.

## Ethics statement

The study was approved by the National Ethics Committee of Slovenia and adhered to Declaration of Helsinki (study registration number: 0120-664/2015-2) approved 11/01/2016. Informed consent was obtained from each patient at inclusion. All methods were performed in accordance with the relevant guidelines and regulations.

## Author contributions

AO, PP, and SB wrote the manuscript and analysed and interpreted the statistical results. AO was a major contributor to writing the manuscript. PP performed the statistical analysis. AO and PP created tables and figures. All authors contributed to the article and approved the submitted version.

## Figures and Tables

**Table:** 

ANOVA	Analysis of variance
ANCOVA	Analysis of covariance
AV	Arterio-venous
BIA	Bioelectrical impedance analysis
BMI	Body mass index
CRP	C-reactive protein
DLM	Dry lean mass
FFMI	Fat-free mass index
HD	Haemodialysis
HGS	Hand-grip strength
IQR	Interquartile range
ISRNM	International Society of Renal Nutrition and Metabolism
ITT	Intention-to-treat
MAC	Mid-arm circumference
MIS	Malnutrition-inflammation score
MUAMC	Mid-upper arm muscle circumference
NUS	Nutritional status
ONS	Oral nutritional supplementation
PEW	Protein-energy wasting
PhA	Phase angle
PP	Per protocol
SD	Standard deviation
SGA	Subjective global assessment
TIBC	Total iron binding capacity
TG	Triglycerides
TSFT	Triceps skinfold thickness
WC	Waist circumference
