# Supplementary material for: Simply adding oral nutritional supplementation to haemodialysis patients may not be enough: a real-life prospective interventional study
Source: Front Nutr. 2023 Oct 19;10:1253164. doi: 10.3389/fnut.2023.1253164 (PMC10620502; doi:10.3389/fnut.2023.1253164)
Supplement: Supplementary file 1 [file Table_1.docx]

Table 1.1: Baseline characteristics - ANOVA

|  | **Group A**  (n=25) | **Group B**  (n=37) | **Group C**  (n=9) | p-value |
| --- | --- | --- | --- | --- |
| ***Demographics*** |  |  |  |  |
| Age (years) | 58.0±13.2 | 65.8±11.3 | 60.3±15.8 | 0.055 |
| Gender (male; n (%)) | 17 (68%) | 19 (51.4%) | 3 (33.3%) | 0.172 |
| Dialysis vintage (months) | 45.4 (19.2-89.7) | 61.9 (30.3-85.7) | 45.7 (44.4-96.0) | 0.868 |
| ***Co-morbidities*** |  |  |  |  |
| Diabetes mellitus | 9 (36%) | 12 (32%) | 3 (33%) | 0.939 |
| Arterial hypertension | 21 (84%) | 32 (86%) | 8 (89%) | 1.000 |
| Cardio-vascular diseases | 5 (23.81%) | 12 (57.14%) | 4 (19.05%) | 0.332 |
| Cancer | 1 (4%) | 10 (27%) | 1 (11%) | **0.043** |
| ***Dietary parameters*** |  |  |  |  |
| Dietary energy intake (kcal/kg/day)  n = 54 | 18.2 (14.0-20.5)  n = 21 | 21.5 (16.2-27.9)  n = 27 | 21.6 (16.8-24.8)  n = 6 | 0.094 |
| Dietary protein intake (g/kg/day)  n = 54 | 0.7 (0.5-1.0)  n = 21 | 0.8 (0.7-1.0)  n = 27 | 0.8 (0.7-1.0)  n = 6 | 0.507 |

Cardiovascular diseases = ischemic heart disease, peripheral arterial disease and cerebral arterial disease. Data are presented n (%), mean ± SD and median (25^th^-75^th^). P values <0.05 were considered statistically significant and are marked bold.
