# Supplementary material for: Simply adding oral nutritional supplementation to haemodialysis patients may not be enough: a real-life prospective interventional study
Source: Front Nutr. 2023 Oct 19;10:1253164. doi: 10.3389/fnut.2023.1253164 (PMC10620502; doi:10.3389/fnut.2023.1253164)
Supplement: Supplementary file 2 [file Table_2.docx]

Table 2.1: Changes in surrogate markers of NUS at baseline and after 12 months - ANOVA

|  |  | **Group A** (n=25) | p-value  (baseline-12 mo) | **Group B** (n=37) | p-value  (baseline-12 mo) | **Group C** (n=9) | p-value  (baseline-12 mo) | p-value  (between groups) |
| --- | --- | --- | --- | --- | --- | --- | --- | --- |
| Albumin (mg/L) | baseline | 42.1±1.6 | **0.015** | 38.1±2.6 | 0.137 | 38.5±2.5 | 0.286 | **<0.001**  **^#^A vs B**  **^#^A vs C** |
|  | 12 mo | 40.0 (38.2-42.1) |  | 38.4 (35.6-39.8) |  | 36.3 (35.3-39.2) |  | **0.014**  **^#^A vs B**  **^#^A vs C** |
| PhA  (◦) | baseline | 5.5±1.1 | 0.258 | 4.4±0.8 | **0.028** | 4.3±1.3 | 0.498 | **<0.001**  **^#^A vs B**  **^#^A vs C** |
|  | 12 mo | 5.2 (4.6-6.1) |  | 4.1 (3.4-4.9) |  | 4.5 (3.1-4.7) |  | **0.001**  **^#^A vs B**  **^#^A vs C** |
| HGS  (kg) | baseline | 24.8±10.2 | 0.115 | 22.1±8.7 | 0.656 | 19.3±7.8 | 0.362 | 0.275 |
|  | 12 mo | 23.7 (17.7-36.7) |  | 21.3 (15.7-27.0) |  | 15.0 (13.7-28.3) |  | 0.119 |

12 mo = after 12 months; PhA = phase angle; HGS = hand grip strength. Data are presented as mean ± SD and median (25^th^-75^th^). P-values <0.05 were considered statistically significant and are marked bold. ^#^ p _(post-hoc)_ < 0.01.
