# Supplementary material for: Simply adding oral nutritional supplementation to haemodialysis patients may not be enough: a real-life prospective interventional study
Source: Front Nutr. 2023 Oct 19;10:1253164. doi: 10.3389/fnut.2023.1253164 (PMC10620502; doi:10.3389/fnut.2023.1253164)
Supplement: Supplementary file 3 [file Table_3.docx]

Table 3.1: Changes in other measured parameters at baseline and after 12 months - ANOVA

|  |  | **Group A** (n=25) | p-value  (baseline-12 mo) | **Group B** (n=37) | p-value  (baseline-12 mo) | **Group C** (n=9) | p-value  (baseline-12 mo) | p-value  (between groups) |
| --- | --- | --- | --- | --- | --- | --- | --- | --- |
| BMI  (kg/m2) | baseline | 27.0 (25.8-30.8) | 0.360 | 24.2 (21.4-28.3) | 0.097 | 21.9 (19.9-23.1) | 0.652 | **0.009**  **^*^A vs B**  **^*^A vs C** |
|  | 12 mo | 27.3 (24.3-29.9) |  | 24.0 (20.7-28.3) |  | 21.9 (19.8-24.8) |  | **0.019**  **^*^A vs C** |
| WC  (cm) | baseline | 103.4 (97.0-108.0) | 0.600 | 93.0 (82.7-107.0) | 0.789 | 89.0 (85.0-93.0) | 0.594 | **0.049**  **^*^A vs C** |
|  | 12 mo | 98.4 (92.0-107.0) |  | 92.5 (83.0-105.5) |  | 90 (83.0-92.0) |  | 0.112 |
| MAC  (cm) | baseline | 31.0 (28.0-33.0) | **0.003** | 27.0 (25.0-31.0) | **0.010** | 26.0 (23.3-28.0) | 0.076 | **0.015**  **^*^A vs B**  **^*^A vs C** |
|  | 12 mo | 29.8 (27.1-31.0) |  | 26.5 (24.6-29.7) |  | 24.5 (21.1-27.0) |  | **0.006**  **^#^A vs C** |
| MUAMC  (cm) | baseline | 26.2±2.5 | **0.001** | 24.4±3.6 | **<0.001** | 22.5±2.7 | **0.008** | **0.008**  **^*^A vs C** |
|  | 12 mo | 24.4±3.1 |  | 22.8±3.6 |  | 20.3±2.6 |  | **0.007**  **^#^A vs C** |
| FFMI | baseline | 18.5±2.7 | **0.039** | 16.2±2.9 | 0.925 | 14.6±2.1 | 0.906 | **<0.001**  **^#^A vs B**  **^#^A vs C** |
|  | 12 mo | 18.5 (16.8-20.7) |  | 16.4 (14.2-18.4) |  | 15.6 (13.1-16.2) |  | **<0.001**  **^#^A vs B**  **^#^A vs C** |
| DLM  (kg) | baseline | 13.3±5.2 | **0.016** | 9.5±4.7 | **<0.001** | 6.9±4.7 | 0.326 | **0.002**  **^*^A vs B**  **^#^A vs C** |
|  | 12 mo | 12.8±5.2 |  | 8.9±4.8 |  | 7.7±4.2 |  | **0.005**  **^*^A vs B**  **^#^A vs C** |
| TIBC | baseline | 47.7 (42.7-52.7) | 0.330 | 42.7 (37.6-47.7) | 0.193 | 42.7 (40.2-45.2) | 0.250 | **0.049** |
|  | 12 mo | 42.7 (39.7-54.2) |  | 41.9 (37.6-45.7) |  | 35.9 (33.6-39.4) |  | 0.081 |
| Total cholesterol (mmol/L) | baseline | 4.2 (3.5-5.0) | 0.679 | 3.9 (3.4-4.7) | 0.827 | 3.6 (3.1-4.8) | 1.000 | 0.463 |
|  | 12 mo | 4.3±0.8 |  | 4.0±1.0 |  | 4.3±1.1 |  | 0.488 |
| TG  (mmol/L) | baseline | 1.7 (1.1-2.5) | 0.594 | 1.4 (1.0-1.9) | 0.700 | 1.2 (0.9-1.6) | 0.800 | 0.128 |
|  | 12 mo | 1.8 (1.1-2.3) |  | 1.4 (1.1-1.6) |  | 1.4 (0.9-1.6) |  | 0.273 |
| CRP  (mg/L) | baseline | 1.0 (1.0-7.0) | 0.224 | 4.0 (1.0-8.0) | 0.122 | 5.0 (3.0-8.0) | 0.151 | 0.557 |
|  | 12 mo | 6.0 (1.0-9.0) |  | 6.0 (1.0-15.0) |  | 13.0 (1.0-22.0) |  | 0.580 |
| Potassium (mmol/L) | baseline | 4.7 (4.3-4.9) | 0.696 | 4.7 (4.5-4.9) | 0.700 | 4.8 (4.5-5.1) | **0.042** | 0.934 |
|  | 12 mo | 4.7±0.7 |  | 4.8±0.7 |  | 4.9±0.5 |  | 0.517 |
| Phosphate (mmol/L) | baseline | 1.7±0.4 | 0.737 | 1.5±0.4 | 0.446 | 1.5±0.4 | 0.820 | 0.058 |
|  | 12 mo | 1.7 (1.4-2.0) |  | 1.4 (1.2-1.6) |  | 1.7 (1.4-1.9) |  | 0.092 |

12 mo = after 12 months; BMI = body mass index; WC = waist circumference; MAC = mid-arm circumference; MUAMC = mid-upper arm muscle circumference; FFMI = fat free mass index; DLM = dry lean mass; TIBC = total iron binding capacity; TG = triglycerides; CRP = C-reactive protein. Data are presented as mean ± SD and median (25^th^-75^th^). P-values <0.05 were considered statistically significant and are marked bold. *p _(post-hoc)_ < 0.05. ^#^ p _(post-hoc)_ < 0.01.
