# Supplementary material for: Simply adding oral nutritional supplementation to haemodialysis patients may not be enough: a real-life prospective interventional study
Source: Front Nutr. 2023 Oct 19;10:1253164. doi: 10.3389/fnut.2023.1253164 (PMC10620502; doi:10.3389/fnut.2023.1253164)
Supplement: Supplementary file 4 [file Table_4.docx]

Table 3.2: Changes in other measured parameters at baseline and after 12 months - ITT

|  |  | Group A (n=25) | Group B* (n=46) | p-value (between groups) | Group A (Adjusted Mean) | Group B (Adjusted Mean) | ANCOVA p-value | Partial Eta Squared for Group (measurement) |
| --- | --- | --- | --- | --- | --- | --- | --- | --- |
| BMI (kg/m2) | baseline | 27.0 (25.8-30.8) | 23.7 (21.2-28.2) | **0.038** | 25.9 | 25.9 | 0.929 | 0.000 (0.962) |
|  | 12 mo | 27.3 (24.3-29.9) | 23.6 (20.6-28.0) | 0.073 |  |  |  |  |
| WC (cm) | baseline | 103.4 (97.0-108.0) | 92.0 (82.8-105.8) | **0.021** | 97.0 | 97.9 | 0.614 | 0.004 (0.883) |
|  | 12 mo | 98.4 (92.0-107.0) | 91.5 (83.0-105.1) | 0.061 |  |  |  |  |
| MAC (cm) | baseline | 31.0 (28.0-33.0) | 27.0 (24.9-30.6) | **0.007** | 27.5 | 27.7 | 0.760 | 0.001 (0.767) |
|  | 12 mo | 29.8 (27.1-31.0) | 26.4 (24.0-29.5) | **0.007** |  |  |  |  |
| MUAMC (cm) | baseline | 26.2±2.5 | 24.0±3.5 | **0.008** | 23.2 | 22.9 | 0.668 | 0.003 (0.693) |
|  | 12 mo | 24.4±3.1 | 22.3±3.5 | **0.014** |  |  |  |  |
| FFMI | baseline | 18.5±2.7 | 15.9±2.8 | **<0.001** | 17.4 | 16.8 | **0.015** | 0.083 (0.905) |
|  | 12 mo | 19.1±2.8 | 15.9±2.8 | **<0.001** |  |  |  |  |
| DLM (kg) | baseline | 13.3±5.2 | 9.0±4.7 | **<0.001** | 10.2 | 10.2 | 0.980 | 0.000 (0.976) |
|  | 12 mo | 12.8±5.2 | 8.7±4.7 | **0.001** |  |  |  |  |
| TIBC | baseline | 47.7 (42.7-52.7) | 42.7 (37.6-47.7) | **0.015** | 47.0 | 46.9 | 0.941 | 0.000 (0.67) |
|  | 12 mo | 45.7 (41.9-55.2) | 44.9 (40.2-47.9) | 0.283 |  |  |  |  |
| Total cholesterol (mmol/L) | baseline | 4.2 (3.5-5.0) | 3.9 (3.3-4.7) | 0.219 | 4.0 | 4.0 | 0.923 | 0.000 (0.516) |
|  | 12 mo | 4.3±0.8 | 4.1±1.0 | 0.310 |  |  |  |  |
| TG (mmol/L) | baseline | 1.7 (1.1-2.5) | 1.4 (0.9-1.9) | 0.051 | 1.8 | 1.5 | 0.067 | 0.049 (0.324) |
|  | 12 mo | 1.8 (1.1-2.3) | 1.4 (1.0-1.6) | 0.107 |  |  |  |  |
| CRP (mg/L) | baseline | 1.0 (1.0-7.0) | 4.5 (1.0-8.0) | 0.313 | 6.7 | 6.7 | 0.957 | 0.000 (0.474) |
|  | 12 mo | 6.0 (1.0-9.0) | 6.5 (1.0-19.0) | 0.444 |  |  |  |  |
| Potassium (mmol/L) | baseline | 4.7 (4.3-4.9) | 4.7 (4.5-5.0) | 0.800 | 4.7 | 4.8 | 0.243 | 0.020 (0.291) |
|  | 12 mo | 4.7±0.7 | 4.8±0.6 | 0.378 |  |  |  |  |
| Phosphate (mmol/L) | baseline | 1.7±0.4 | 1.5±0.4 | **0.018** | 1.6 | 1.5 | 0.395 | 0.011 (0.24) |
|  | 12 mo | 1.7±0.4 | 1.5±0.5 | 0.061 |  |  |  |  |

12 mo = after 12 months; BMI = body mass index; WC = waist circumference; MAC = mid-arm circumference; MUAMC = mid-upper arm muscle circumference; FFMI = fat free mass index; DLM = dry lean mass; TIBC = total iron binding capacity; TG = triglycerides; CRP = C-reactive protein. Data are presented as mean ± SD and median (25^th^-75^th^). P-values <0.05 were considered statistically significant and are marked bold.
