# Supplementary material for: Simply adding oral nutritional supplementation to haemodialysis patients may not be enough: a real-life prospective interventional study
Source: Front Nutr. 2023 Oct 19;10:1253164. doi: 10.3389/fnut.2023.1253164 (PMC10620502; doi:10.3389/fnut.2023.1253164)
Supplement: Supplementary file 5 [file Table_5.docx]

Table 3.3: Changes in other measured parameters at baseline and after 12 months – PP

|  |  | Group A (n=25) | Group B (n=37) | p-value (between groups) | Group A (Adjusted Mean) | Group B (Adjusted Mean) | ANCOVA p-value | Partial Eta Squared for Group (measurement) |
| --- | --- | --- | --- | --- | --- | --- | --- | --- |
| BMI (kg/m2) | baseline | 27.0 (25.8-30.8) | 24.2 (21.4-28.3) | **0.012** | 26.3 | 26.3 | 0.906 | 0.000 (0.962) |
|  | 12 mo | 27.3 (24.3-29.9) | 24.0 (20.7-28.3) | **0.018** |  |  |  |  |
| WC (cm) | baseline | 103.4 (97.0-108.0) | 93.0 (82.7-107.0) | 0.055 | 98.0 | 99.0 | 0.571 | 0.005 (0.871) |
|  | 12 mo | 98.4 (92.0-107.0) | 92.5 (83.0-105.5) | **0.036** |  |  |  |  |
| MAC (cm) | baseline | 31.0 (28.0-33.0) | 27.0 (25.0-31.0) | **0.018** | 27.7 | 28.3 | 0.212 | 0.026 (0.849) |
|  | 12 mo | 29.8 (27.1-31.0) | 26.5 (24.6-29.7) | **0.036** |  |  |  |  |
| MUAMC (cm) | baseline | 26.2±2.5 | 24.4±3.6 | **0.022** | 23.4 | 23.4 | 0.908 | 0.000 (0.679) |
|  | 12 mo | 24.4±3.1 | 22.8±3.6 | **0.069** |  |  |  |  |
| FFMI | baseline | 18.5±2.7 | 16.2±2.9 | **0.002** | 17.8 | 17.1 | **0.018** | 0.091 (0.899) |
|  | 12 mo | 19.1±2.8 | 16.2±2.9 | **<0.001** |  |  |  |  |
| DLM (kg) | baseline | 13.3±5.2 | 9.5±4.7 | **0.004** | 10.5 | 10.4 | 0.801 | 0.001 (0.975) |
|  | 12 mo | 12.8±5.2 | 8.9±4.8 | **0.004** |  |  |  |  |
| TIBC | baseline | 47.7 (42.7-52.7) | 42.7 (37.7-47.7) | **0.026** | 47.4 | 47.4 | 0.989 | 0.000 (0.684) |
|  | 12 mo | 45.7 (41.9-55.2) | 44.9 (42.7-47.7) | 0.472 |  |  |  |  |
| Total cholesterol (mmol/L) | baseline | 4.2 (3.5-5.0) | 3.9 (3.4-4.7) | 0.266 | 4.0 | 4.0 | 0.923 | 0.000 (0.516) |
|  | 12 mo | 4.3±0.8 | 4.0±1.0 | 0.242 |  |  |  |  |
| TG (mmol/L) | baseline | 1.7 (1.1-2.5) | 1.4 (1.0-1.9) | 0.078 | 1.8 | 1.5 | 0.127 | 0.039 (0.343) |
|  | 12 mo | 1.8 (1.1-2.3) | 1.4 (1.1-1.6) | 0.111 |  |  |  |  |
| CRP (mg/L) | baseline | 1.0 (1.0-7.0) | 4.0 (1.0-8.0) | 0.399 | 6.7 | 6.6 | 0.937 | 0.000 (0.535) |
|  | 12 mo | 6.0 (1.0-9.0) | 6.0 (1.0-15.0) | 0.591 |  |  |  |  |
| Potassium (mmol/L) | baseline | 4.7 (4.3-4.9) | 4.7 (4.5-4.9) | 0.880 | 4.7 | 4.8 | 0.416 | 0.011 (0.265) |
|  | 12 mo | 4.7±0.7 | 4.8±0.7 | 0.534 |  |  |  |  |
| Phosphate (mmol/L) | baseline | 1.7±0.4 | 1.5±0.4 | **0.029** | 1.7 | 1.5 | 0.29 | 0.019 (0.217) |
|  | 12 mo | 1.7 (1.4-2.0) | 1.4 (1.2-1.6) | **0.032** |  |  |  |  |

12 mo = after 12 months; BMI = body mass index; WC = waist circumference; MAC = mid-arm circumference; MUAMC = mid-upper arm muscle circumference; FFMI = fat free mass index; DLM = dry lean mass; TIBC = total iron binding capacity; TG = triglycerides; CRP = C-reactive protein. Data are presented as mean ± SD and median (25^th^-75^th^). P-values <0.05 were considered statistically significant and are marked bold.
